# N‑Doped Carbon Dot-Based Nanoconjugates with Simultaneous Generation of Nitric Oxide and Singlet Oxygen for Phototherapeutic Applications

**DOI:** 10.1021/acsanm.5c02198

**Published:** 2025-06-16

**Authors:** Francesca Laneri, Cristina Parisi, Vittoria Andrigo, Juliana Guerra Pinto, Luciana Cortez Marcolino, Juliana Ferreira-Strixino, Marta Maria Natile, Salvatore Sortino

**Affiliations:** ! PhotoChemLab, Department of Drug and Health Sciences, 9298 University of Catania, Catania I-95125, Italy; @ ICMATE-CNR Institute of Condensed Matter Chemistry and Technologies for Energy, National Research Council and Department of Chemical Science, 119644 University of Padova, Padova 35131, Italy; # Laboratory of Photobiology Applied to Health, Research and Development Institute, University of Vale do Paraíba, Urbanova I-2911, Brazil

**Keywords:** light, carbon dots, nitric oxide, singlet oxygen, photodynamic therapy

## Abstract

Nitric oxide (NO) and singlet oxygen (^1^O_2_) represent two of the most intriguing agents for unconventional phototherapeutic applications in cancer. In this contribution, N-doped carbon dots (**NCDs**) with strong absorption in the biocompatible green region have been synthesized and covalently decorated with an otherwise blue-light-activatable NO photodonor (NOPD), leading to a nanoconjugate *ca.* 3.5 nm in diameter. The **NCD** core of the nanoconstruct acts as a green light antenna, permitting the release of NO from the NOPD by an intramolecular photoinduced electron transfer, with an improvement of more than 100 nm in the excitation wavelength. Simultaneously, green light excitation generates ^1^O_2_ by collisional energy transfer with molecular oxygen. Due to its emissive properties, the nanoconjugate can be visualized in 9L/LacZ brain cancer cells, where it localizes mainly in the cytoplasm. Amplified mortality of cancer cells is observed upon green light irradiation due to the mutual photodynamic action of NO and ^1^O_2_.

## Graphical abstract

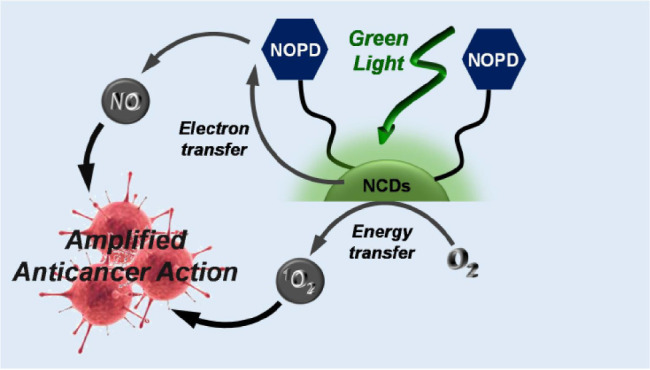

## 1 Introduction

Nitric oxide (NO) and singlet oxygen (^1^O_2_) represent two of the most intriguing unconventional agents for phototherapeutic applications.^1–3^ They combine several common advantages over conventional drug molecules, such as the absence of multidrug resistance, reactivity with all biological components, and, due to their short lifetime, confinement of their region of action to below 200 μm, with reduced systemic effects.

NO plays many physiological and pathophysiological roles,^4,5^ and its use as a therapeutic agent in several diseases including cancer has been extensively demonstrated.^6–13^ However, NO’s effects in cancer strictly depend on its concentration and generation site.^14^ This makes the light-activated NO precursors, namely NO photodonors (NOPDs), very appealing.^15–23^ In NOPDs, the excitation light breaks a covalent bond, uncaging the NO that was initially integrated within their molecular skeleton.

^1^O_2_ plays a dominant role in photodynamic therapy (PDT)^24,25^ and, in contrast to NO, is usually generated in a catalytic fashion by suitable photosensitizers (PS) through collisional energy transfer with nearby molecular oxygen.^26–28^

PSs and NOPDs present the great advantage of not being active in the dark but generating a burst of cytotoxicity exclusively under light inputs in the region of space confined to the irradiated area with superb spatiotemporal control. In this frame, creating single nanoplatforms that combine bimodal phototherapeutic performance and exploit the additive/synergistic effects of simultaneously generated NO and ^1^O_2_ has proven to be innovative in nanomedicine.^29–32^ Our pioneering studies in this regard^33,34^ have inspired the achievement of a number of molecular hybrids and supramolecular nanoconstructs devoted to this goal.^22,23^ Due to their covalent linking, the former ensure that both cytotoxic species are generated in the very same region of space but, on the other hand, present the limitation of a small reservoir of NO compared to the catalytically generated ^1^O_2_ due to the equimolar NOPD:PS molar ratio. The latter offer the advantage of facile tuning of the NOPD:PS molar ratio, but in contrast, these components can diffuse apart due to potential disassembling in a biological environment. On these bases, achieving robust nanostructures covalently integrating multiple NOPD and PS in the same scaffold is challenging since they can overcome the above limitations.

Carbon dots (CDs) are usually spherical carbon nanoparticles exhibiting low toxicity, excellent biocompatibility, and high cell permeability.^35^ They consist of a core rich mainly of sp^2^ hybrid carbons and a shell that can bear different organic functional groups, including amines, hydroxyl, and carboxylic, depending on the precursors used for the synthesis.^36–38^ Besides making CDs well-dispersible in water, these moieties permit surface engineering with additional functional molecular units through simple synthetic protocols.^36–38^ CDs show excitation wavelength-dependent emission, which is helpful for their tracking in a bioenvironment,^39,40^ and can act as both electron/energy donors and acceptors in intra- and interphotoinduced processes with suitable counterparts.^41–43^ This wealth of properties makes these materials intriguing nanoplatforms for various applications in nanomedicine and photonanomedicine.^44–47^ In this regard, we have recently reported on N-doped CDs (**NCDs**) decorated with an NOPD activatable by blue light, demonstrating that blue light excitation amplifies the NO release from the NOPD through an intramolecular photoinduced electron transfer from the **NCDs** core to the peripheral NOPD.^48^

One of the most interesting aspects of CDs relates to their recently demonstrated capability to generate ^1^O_2_ as alternative to the typical PS based on either porphyrinoids or BODIPY derivatives.^49–51^ **NCDs** have been revealed to be more efficient in this regard, with the photosensitization properties strictly dependent on the N doping.^52^

The above scenarios inspired us to achieve an **NCDs**-based nanoconstruct that can simultaneously generate NO and ^1^O_2_ with highly biocompatible green light. To this end, we have devised a novel nanoconjugate **NCDs-1** (Scheme 1). It covalently integrates the NOPD **1**, developed in our group^53,54^ and otherwise activatable by blue light, into **NCDs** exhibiting significant absorption in the green region. We show that the **NCDs** core of the nanoconstruct acts as the sole green light-harvesting antenna and triggers NO generation, probably by a photoinduced electron transfer with NOPD **1** in the shell, and ^1^O_2_ by a bimolecular energy transfer with molecular oxygen. This results in the amplified mortality of cancer cells due to the simultaneous photodynamic action of these two cytotoxic species.

**Scheme 1:**
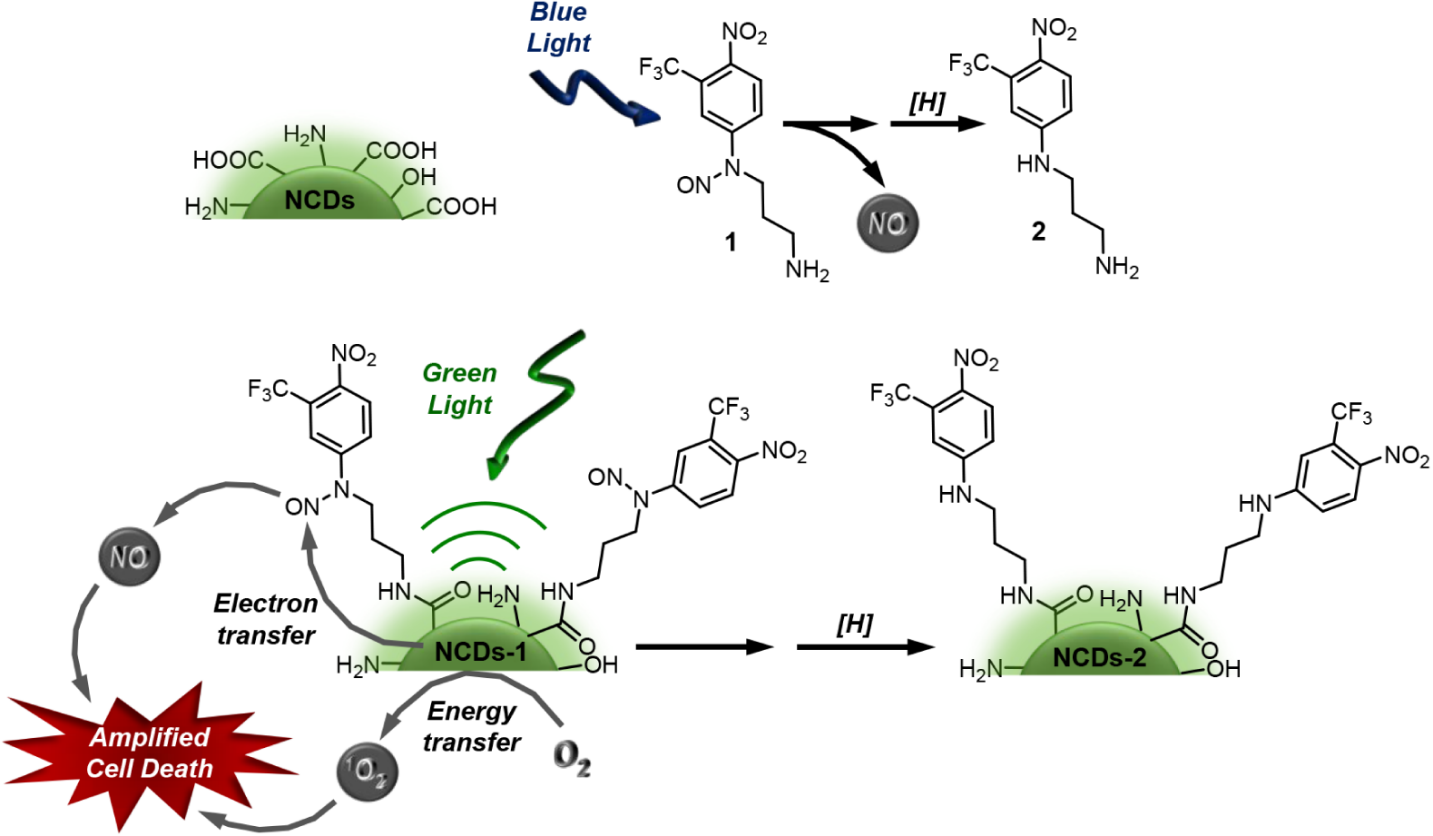
Sketch of the Naked **NCDs**, the NOPD **1** and Its Stable Photoproduct **2** Formed after NO Release under Blue Light, and the Nanoconjugate **NCDs-1** with its Working Principle.

## 2 Experimental Section

### 2.1 Materials and Methods

All chemicals were purchased from Sigma-Aldrich and used as received. All solvents used (Sigma-Aldrich) were of spectrophotometric grade. Deionized ultrafiltered water was used throughout this study.

#### 2.1.1 Synthesis

NOPD **1** was synthesized as previously described.^53,54^ **NCDs** were synthesized *via* an already reported solvothermal method using citric acid and urea, with dimethylformamide (DMF) as the solvent,^55^ with some modifications. Specifically, 1 g of citric acid was reacted with 2 g of urea at 160 °C for 6 h in 10 mL of DMF. After cooling at room temperature, the resulting dark red solution was treated with 20 mL of an aqueous NaOH solution (50 mg mL^– 1^) and stirred for 1 min. The obtained solution was dialyzed overnight and later freeze-dried to give a dark purple product. 100 mg of this product was then solubilized in 20 mL of an aqueous HCl solution (5 wt %) and stirred for 10 min to remove surface metal cations. The solution was then centrifuged at 14,000 rpm for 10 min, and the solid was collected, solubilized in water, and centrifuged again twice at 14,000 rpm for 10 min, to eliminate residual salts and HCl. The final product was freeze-dried, yielding a dark powder of **NCDs**.

**NCDs-1** were prepared as follows: To a 3 mL aqueous dispersion containing 15 mg of **NCDs**, 144 mg of 1-ethyl-3-(3-(dimethylamino)­propyl)­carbodiimide (EDC) and 86 mg of *N*-hydroxysuccinimide (NHS) were added. The mixture was sonicated for 15 min in an ice bath. Subsequently, 1 mL of an acetonitrile solution containing 45 mg of compound **1** was added, and the reaction mixture was stirred for 2 days in the dark at room temperature. Afterward, the acetonitrile was evaporated under vacuum, and the resulting product was dialyzed overnight and freeze-dried, yielding **NCDs-1**. Based on the molar absorptivity of compound **1** at 290 nm (9,600 M^–1^ cm^–1^), a functionalization degree of *ca.* 70% can be estimated.

#### 2.1.2 Fluorescence, NO, and ^1^O_2_ Quantum Yields

Fluorescence quantum yields (Φ_f_) were determined at λ_exc_ = 530 nm using Rhodamine 6G in EtOH (Φ_f_ = 0.96) as the standard.^56^

NO photogeneration quantum yield (Φ_NO_) was determined at λ_exc_ = 532 nm, according to our reported procedure.^48^

^1^O_2_ quantum yields (Φ_Δ_) were determined at λ_exc_ = 532 nm in D_2_O (1% MeOD) using Rose Bengal as the standard (Φ_Δ_ = 0.76).^56^

### 2.2 Cell Experiments

Gliosarcomas of the 9L/LacZ lineage obtained from the Rio de Janeiro Cell Bank were maintained in Dulbecco’s Modified Eagle’s Medium (DMEM) (Gibco), supplemented with 10% fetal bovine serum (FBS) (LGC Biotechnology) and 1% penicillin/streptomycin solution (LGC Biotechnology), and kept in an incubator at 37 °C with 5% CO_2_.

#### 2.2.1 Internalization

Initially, 10^6^ cells were adhered to glass coverslips disseminated in 24-well plates and left to incubate for 24 h in an incubator at 37 °C with 5% CO_2_. The culture medium was then removed, and **NCDs-1** was added and incubated for 1 h, to allow the compound to be internalized, followed by washing with PBS and fixation with 4% paraformaldehyde for 15 min at room temperature. The slides were mounted with ProLong Diamond Antifade Mountant with DAPI (Thermo Fisher) and analyzed under a Zeiss LSM 700 confocal microscope. The groups were protected from light during the process.

#### 2.2.2 Cell Viability

10^5^ cells were transferred in two 96-well plates, one plate was irradiated, and the other to remained in the dark. After cell adhesion, the medium was removed, and either **NCDs** or **NCD-1** were added. For the groups without the compound, the same volume of PBS was added, and the plates were then irradiated in Biotable (Biotable PhotoBioS) for 48 min (530 nm, 83 mW/cm^2^). The Biotable Photobios Green—Model V1 (Photobios, Brazil) is a custom-designed photobiological irradiation platform equipped with 12 high-power LEDs (10 W each) emitting green light at a wavelength of 520 nm. The system provides a calibrated irradiance of 83.33 mW/cm^2^ over a uniform 11 × 16 cm active area, ensured by the fixed arrangement of the LEDs at 1 cm from the irradiation plane and a 120° emission angle per diode. After irradiation, the PBS and compounds were removed from the plates, and DMEM medium was added. The plates were incubated at 37 °C and 5% CO_2_ for 24 h. The experiment was performed in quadruplicate and protected from light during the process. After the respective treatments, viability tests were performed using the rypan blue assay. This method allows for the differentiation of live cells from dead cells by observing cell coloration. The procedure was performed 24 h after the application of treatment, with a 0.2% Trypan Blue solution (Sigma), and incubated for 5 min. After this time, the solution was removed, and PBS was added. The groups were analyzed using a Zeiss Axio Vert.A1 inverted microscope.

#### 2.2.3 Statistical analysis

Statistical analyses were performed using GraphPad software, version 7.04 (GraphPad Software, Inc., La Jolla, CA, USA). Statistically significant comparisons between the experimental groups and the control group were presented, considering results with *p* < 0.001 as significant.

### 2.3 Instrumentations

HRTEM images were acquired with a previously described microscope.^48^

XPS measurements were performed with an ESCALAB QXi spectrometer from Thermo Fisher Scientific, using a monochromatic Al Kα source (1486.6 eV) operating at 200 W and a spot size of 650 μm × 200 μm.

The instrumentation for FTIR, UV–vis absorption and emission spectra, and time-resolved fluorescence has been previously described.^48^

Steady-state irradiation experiments were performed according to already reported setup^48^ using a CW green laser (200 mW) at λ = 532 nm or, in the case of TPPS, with a LED at 420 nm (10 mW).

NO and ^1^O_2_ were detected by a direct method exploiting an ultrasensitive NO electrode and the typical NIR luminescence of ^1^O_2_, respectively, as previously reported.^48,32^

## 3 Results and Discussion

HRTEM micrograph of **NCDs** reveals quite dispersed, spherical-like shaped **NCDs** with a mean diameter of 3.3 ± 0.1 nm (Figure 1). The **NCDs** are crystalline, as indicated by the well-visible lattice fringes. The interplanar spacing of 0.21 nm accords with that observed in graphene sheets.^57^

**Figure 1:**
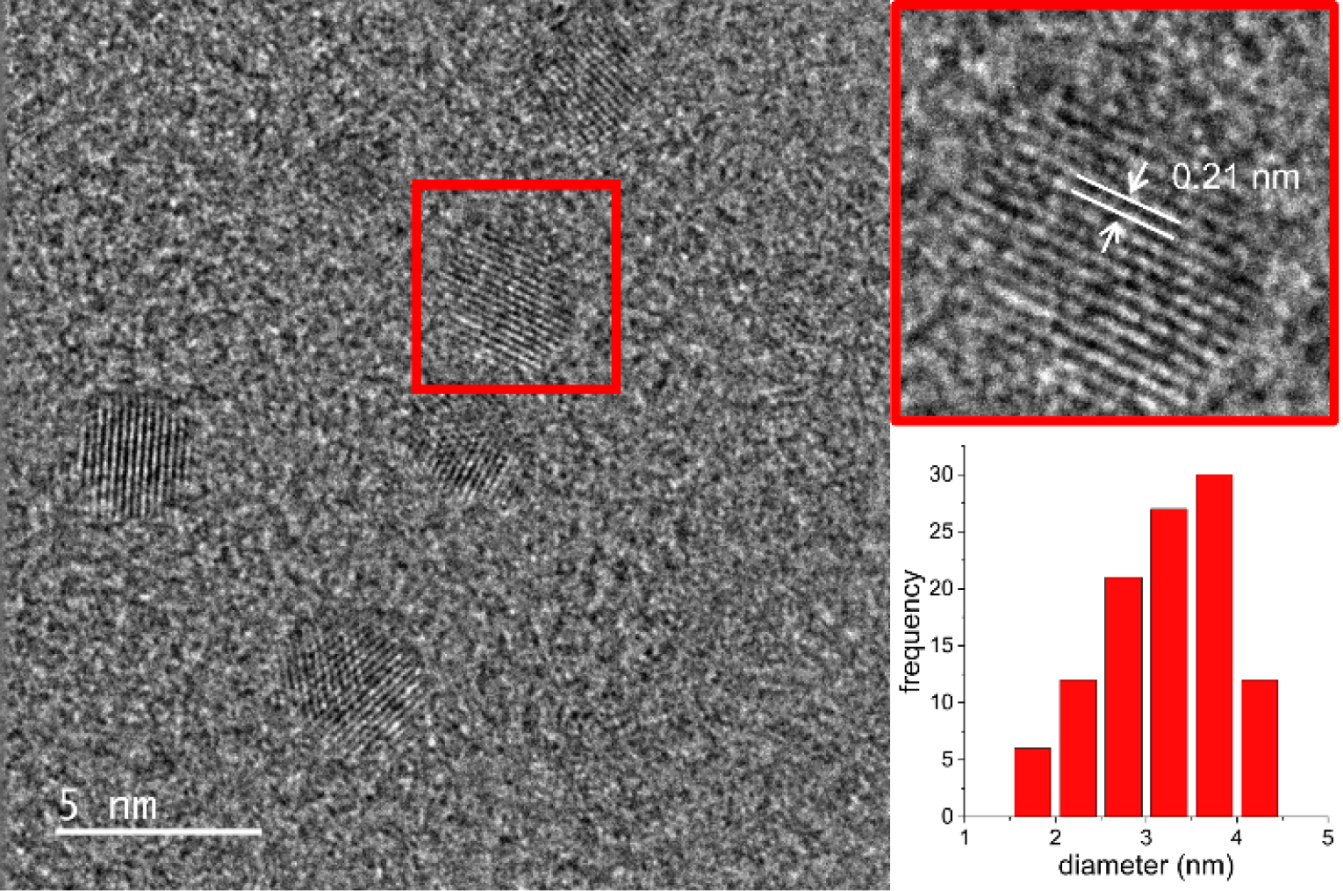
Representative HRTEM images and size distribution of **NCDs**.

The XPS analysis of **NCDs** reveals the presence of C (60.4 at%), N (24 at%), O (11.9 at%), and traces of Na (3.8 at%). Careful analysis of the C 1s, N 1s, and O 1s high-resolution spectra provided information on the chemical environment of different species (Figure 2A–C).

**Figure 2:**
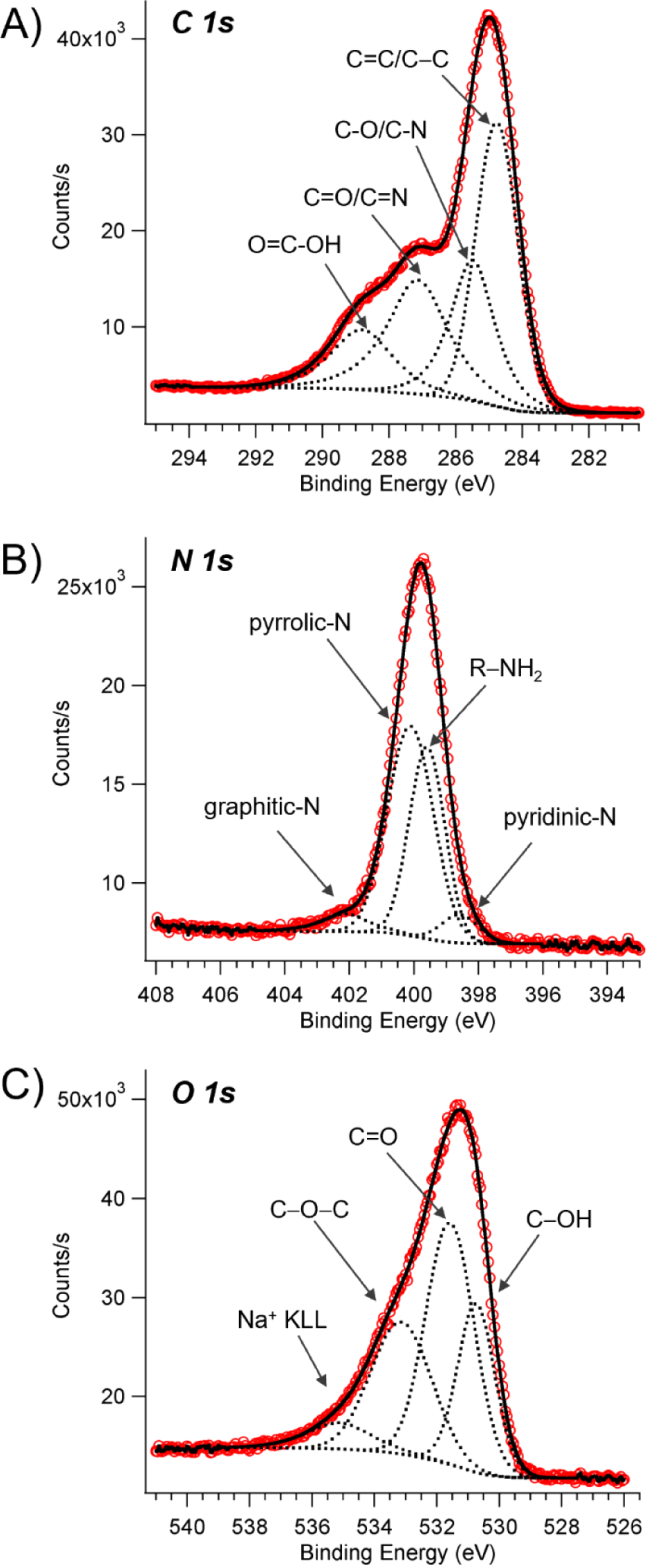
XPS analysis of **NCDs**,: high resolution spectra of (A) C 1s, (B) N 1s, and (C) O 1s. Red circles for raw data; black continuum line and dashed lines for the fitting curves.

The deconvolution of the C 1s core level reveals the presence of four different carbon species at 284.8 eV (C=C, C–C), 285.5 eV, 287.1 eV (C=O, C=N), and 288.8 eV (O=C–OH), respectively.^58,59^ The N 1s spectrum can be deconvoluted into four peaks with maxima at 398.6 eV, 399.6 eV, 400.1 eV, and 402.1 eV, consistent with pyridinic-N, aminic R-NH_2_, pyrrolic-N, and graphitic-N, respectively.^60^ The O 1s region consists of three contributions at 530.7 eV, 531.6 eV, and 533.1 eV, ascribable to C–OH, C=O, and O–C–O. The small peak at 535.1 eV is due to KLL of Na^+^.^60^ The atomic percentages of different species are shown in Table S1.

FTIR analysis of **NCDs** (Figure 3) showed the presence of a broad band ranging from *ca.* 3700 cm^–1^ to 2900 cm^–1^, attributed to the typical stretching frequency of −OH, −NH, and −COOH groups localized on the surface scaffolds, responsible for the hydrophilic nature of the nanostructures,^61^ which resulted in them being well dispersible in water and suitable for further functionalization. The band at 1555 cm^–1^ was related to C=C stretching, and the distinctive band at 1700 cm^–1^ was related to the C=O stretching of the carboxylic groups. FTIR analysis also proved the successful grafting of compound **1** at the **NCDs** shell (Figure 3). The peak related to the C=O stretching shifted to 1632 cm^–1^ in **NCDs-1**, in good agreement with the formation of an amide bond. In contrast, the stretching vibration of −R–N=O at 1540 cm^–1^ in the case of 1 ^62^ was unaltered in the nanoconjugate. HRTEM analysis of **NCDs-1** revealed no significant changes in size and morphology with respect to **NCDs** (Figure S1). **NCDs-1** were well-dispersible in water medium, remaining stable for *ca.* one month with negligible evidence of aggregation (confirmed by the unchanged absorption spectrum, see below).

**Figure 3:**
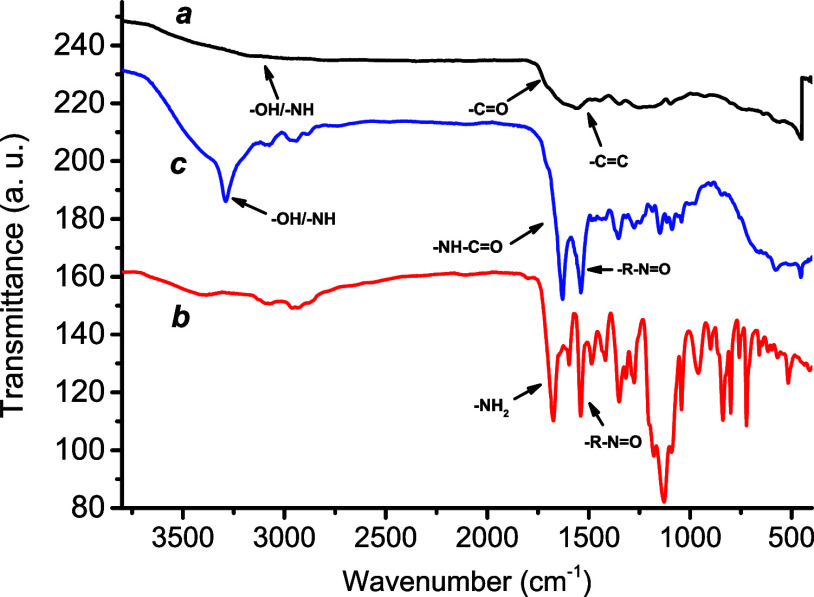
FTIR spectra of **NCDs** (a), **1** (b), and **NCDs-1** (c).

Compound **1** exhibits an absorption maximum at 290 nm and a tail extending beyond 400 nm (Figure S2). Irradiation of compound **1** with blue light induces the homolytic rupture of the N–NO bond, decaging of NO, and formation of the stable photoproduct **2** after H transfer from the solvent to the anilinyl radical intermediate (see Scheme 1).^53,54^ The stable photoproduct **2** exhibits molar absorptivity similar to compound **1**, but its absorption maximum is significantly shifted to longer wavelengths (*ca.* 100 nm) due to the push–pull character of the nitroaniline moiety (Figure S2).^53,54,63,64^

Figure 4A shows the absorption spectra of **NCDs-1** and, for the sake of comparison, those of **NCDs**, compound **1**, and their mixture. **NCDs-1** exhibits the fingerprints of both components with an intense UV band at *ca.* 300 nm, typical of compound **1**, and the broad absorption of **NCDs** in the green spectral window at *ca.* 560 nm. Besides, a new absorption is present in the interval of 400–500 nm. These absorption features differ from those of the mixture of the two components (see spectrum *d* in Figure 4A) and account for a remarkable interaction between compound **1** and the **NCDs** scaffold of the nanoconjugate in the ground state. On the basis of the electron-accepting and electron-donating features of compound **1** and **NCDs**, respectively, the new absorption in the region of 400–500 nm is probably charge transfer in character. This is in agreement to what already found in our recent work for the same NOPD grafted onto UV-absorbing **NCDs** ^48^ and is supported by the study of Guldi and coworkers in the case of nanoconjugates of CDs integrating electron-acceptor chromophoric components.^65^

**Figure 4:**
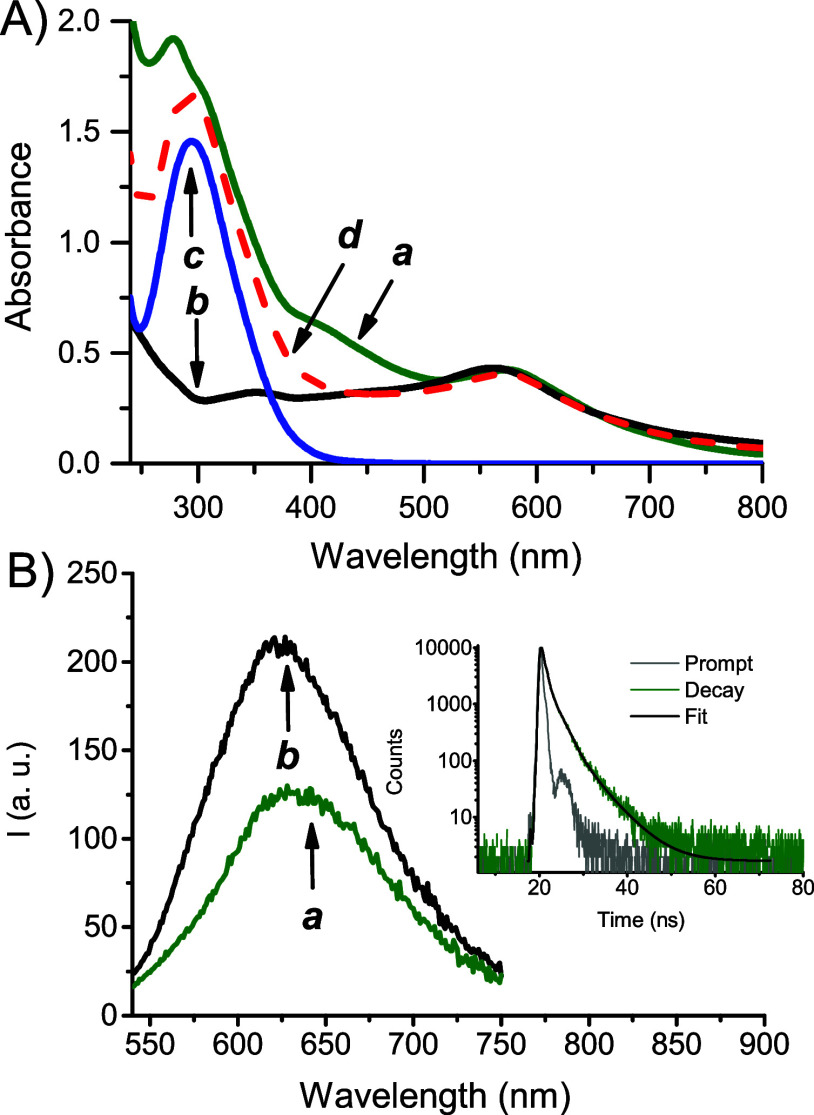
(A) Absorption spectra of aqueous dispersions (1% MeOH) of **NCDs-1** (70 μg mL^–1^) (a), **NCDs** (17 μg mL^–1^) (b), **1** (53 μg mL^–1^) (c), and the mixture of **NCDs** + **1** (d). (B) Fluorescence emission spectra of aqueous dispersions (1% MeOH) of **NCDs-1** (70 μg mL^–1^) (a) and **NCDs** (17 μg mL^–1^) (b) at λ_exc_ = 530 nm, *T* = 25 °C. The inset shows the fluorescence decay and the related triexponential fitting of the **NCDs**.

As typically observed in different types of CDs,^61^ **NCDs** show emission dependent on the excitation wavelength (Figure S3) and, in this case, it mainly falls in the green/red region. Figure 4B shows that the emission of **NCDs** (Φ_f_ = 0.03) is partially quenched in **NCDs-1** (Φ_f_ = 0.018). These findings account for an interaction between the components in the excited state. Regarding the quenching observed, energy transfer between the **NCDs** core and the peripheral **1** is out of the question. The emission of the former (energy donor) falls, in fact, well beyond the absorption of the latter (energy acceptor) (absence of spectral overlap), making this process thermodynamically unfeasible. Instead, we believe that analogously to what was observed in our previous work,^48^ a photoinduced electron transfer from **NCDs** to the strong electron acceptor **1** is more likely. Note that the emission dynamics of **NCDs** exhibit a triexponential decay, typical for CDs,^39,40^ with lifetimes (τ) and related amplitudes (α) being τ_1_ = 4.87 ns (α_1_ = 12.36%), τ_2_ = 1.8 ns (α_2_ = 39.22%), and τ_3_ = 0.29 ns (α_3_ = 48.41%) (inset Figure 4B) and attributable to diverse fluorophoric domains.^39,40^ These values did not significantly change in **NCDs-1**, suggesting that the quenching of the emission takes place on a time scale below our time resolution (*ca.* 200 ps). This hypothesis is in line to what is already found in CD nanoconjugates functionalized with strong electron acceptor chromophoric components, in which quenching by photoinduced electron transfer occurring on a ps time scale was observed by ultrafast spectroscopy.^65^

Irradiation of **NCDs-1** with green light under anaerobic conditions shows that the absorption of the **NCDs** core beyond 560 nm remains almost unaltered, whereas a new absorption band is observed at *ca.* 400 nm (Figure 5A). The growth of this band is also observed under aerobic conditions, but in this case, bleaching of the **NCDs** core absorption is noted (Figure 5B). The kinetic profile at 400 nm is very similar in the absence or presence of oxygen (Figure 5C). Note that the new band at ca. 400 nm is the same as that observed for the unbound **1** under blue light excitation^48^ and is due to the nitroaniline-based chromophoric motif with push–pull character, typical of compound **2** (see Scheme 1). Therefore, the photolysis profile of **NCDs-1** accounts for the NO detachment stimulated by green light, leading to **NCDs-2** as the more likely stable photoproduct (see Scheme 1). The value for the quantum yield Φ_NO_ can be estimated to be 0.9 (± 0.1) × 10^–3^. NO photogeneration was unambiguously confirmed by its direct detection by an amperometric technique upon light and dark alternate cycles (Figure 5D). Since compound **1** does not show any absorption beyond 450 nm (see *c* in Figure 4A Figure 2), the NO photorelease from **NCDs-1** under green light excitation cannot be due to the direct absorption of this component, which is unreactive under these conditions (Figure S4). We believe that the photoinduced electron transfer from **NCDs** to compound **1** can be reasonably responsible for NO uncaging. Such a mechanism is not uncommon for *N*-nitroso derivatives and involves the radical anion centered on the nitroso group, which is characterized by a low bond enthalpy compared to the N–NO neutral form, encouraging fast NO detachment.^66^ A similar mechanism was recently proposed for nitroso derivatives with the same or similar chromophoric group photostimulated by appropriate photosensitizers.^67,68^ The proposed mechanism is also in excellent agreement with the negligible effect of oxygen on the photolysis kinetics (Figure 5C). In fact, the very short emission lifetimes of **NCDs-1** (see above) rule out any potential bimolecular quenching by oxygen, even at diffusional rates. As the introductory part outlines, analogously to typical organic and metallo-organic PS, *ad hoc* prepared CDs generate ^1^O_2_ by energy transfer via the Dexter mechanism.^49–51^ In our case, both direct and indirect measurements demonstrate ^1^O_2_ photogeneration upon excitation of **NCDs-1** with green light. Figure 6A shows that irradiation of **NCDs-1** leads to the unambiguous generation of ^1^O_2_, as revealed by its characteristic luminescence spectrum in the near-IR spectral region (maximum at *ca.* 1270 nm).^25^ The value for the quantum yield Φ_Δ_ can be estimated to be 0.08 ± 0.01. In this view, the bleaching of the absorption at 560 nm related to the **NCDs** core of **NCDs-1** observed in the photolysis experiments exclusively under aerobic conditions (see Figure 5B) might reasonably be due to partial self-oxidation of the sp^2^ of the **NCDs-1** core by the photogenerated ^1^O_2_. This hypothesis is supported by literature data demonstrating the scavenging properties of CDs toward reactive oxygen species^69,70^ and confirmed by photolysis experiments performed in the presence of histidine, a typical ^1^O_2_ quencher. As shown in Figure 6B, no bleaching of the absorption belonging to the **NCDs** core was observed under aerobic conditions due to the competition of the quencher with the **NCDs** core in the bimolecular reaction was performed with ^1^O_2_. On the other hand, the quencher did not affect the photorelease of NO, as proven by the formation of the characteristic band at 400 nm, indicative of the denitrosation of **NCDs-1**. An additional proof demonstrating the involvement of the photogenerated ^1^O_2_ in the partial self-oxidation of **NCDs-1** was provided by photolysis experiments carried out with excitation light at 420 nm in the presence of the hydrosoluble 5,10,15,20-tetrakis­(4-sulfonatophenyl)-21*H*,23*H*-porphyrin (TPPS), an efficient ^1^O_2_ PS.^56^ At this excitation wavelength, **NCDs-1** does not show significant bleaching (Figure S5), in line with the lack of ^1^O_2_ production. In contrast, ^1^O_2_ is effectively generated by TPPS that absorbs almost exclusively the excitation light due to its intense Soret band. As illustrated in Figure 6C, significant bleaching of the visible band of **NCDs-1** at 560 nm was observed after only a few seconds of irradiation.

**Figure 5:**
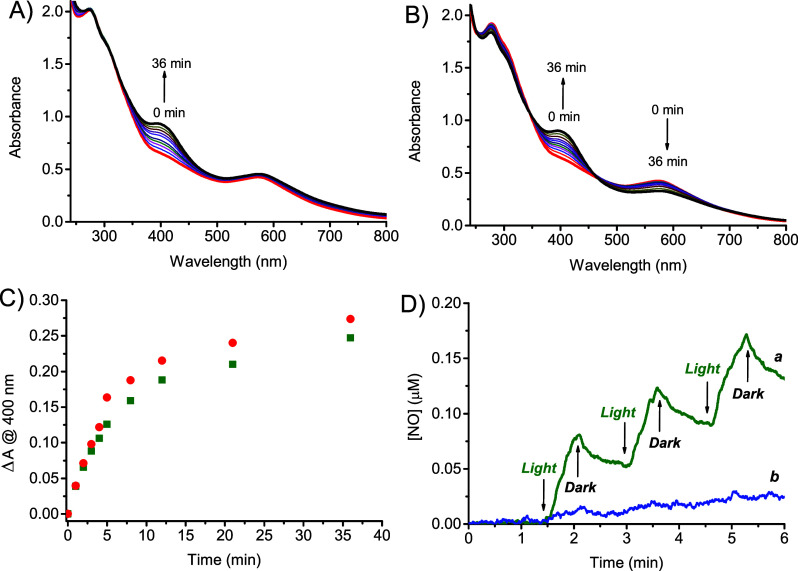
Absorption spectral changes observed under 532 nm light excitation of N_2_-saturated (A) and air-equilibrated (B) aqueous dispersions (1% MeOH) of **NCDs-1** (70 μg mL^–1^) at different irradiation times (from 0 to 36 min). The arrows indicate the evolution of the spectral profile with the illumination time. (C) Difference of absorbance observed at 400 nm for **NCDs-1** related to the photolysis as in (A) (●) and (B) (■). (D) NO release profile observed for air-equilibrated aqueous dispersions (1% MeOH) of **NCDs-1** (70 μg mL^–1^) (a) and, for comparison, **NCDs** (17 μg mL^–1^) (b), upon alternate cycles of irradiation at λ_exc_ = 532 nm, *T* = 25 °C.

**Figure 6:**
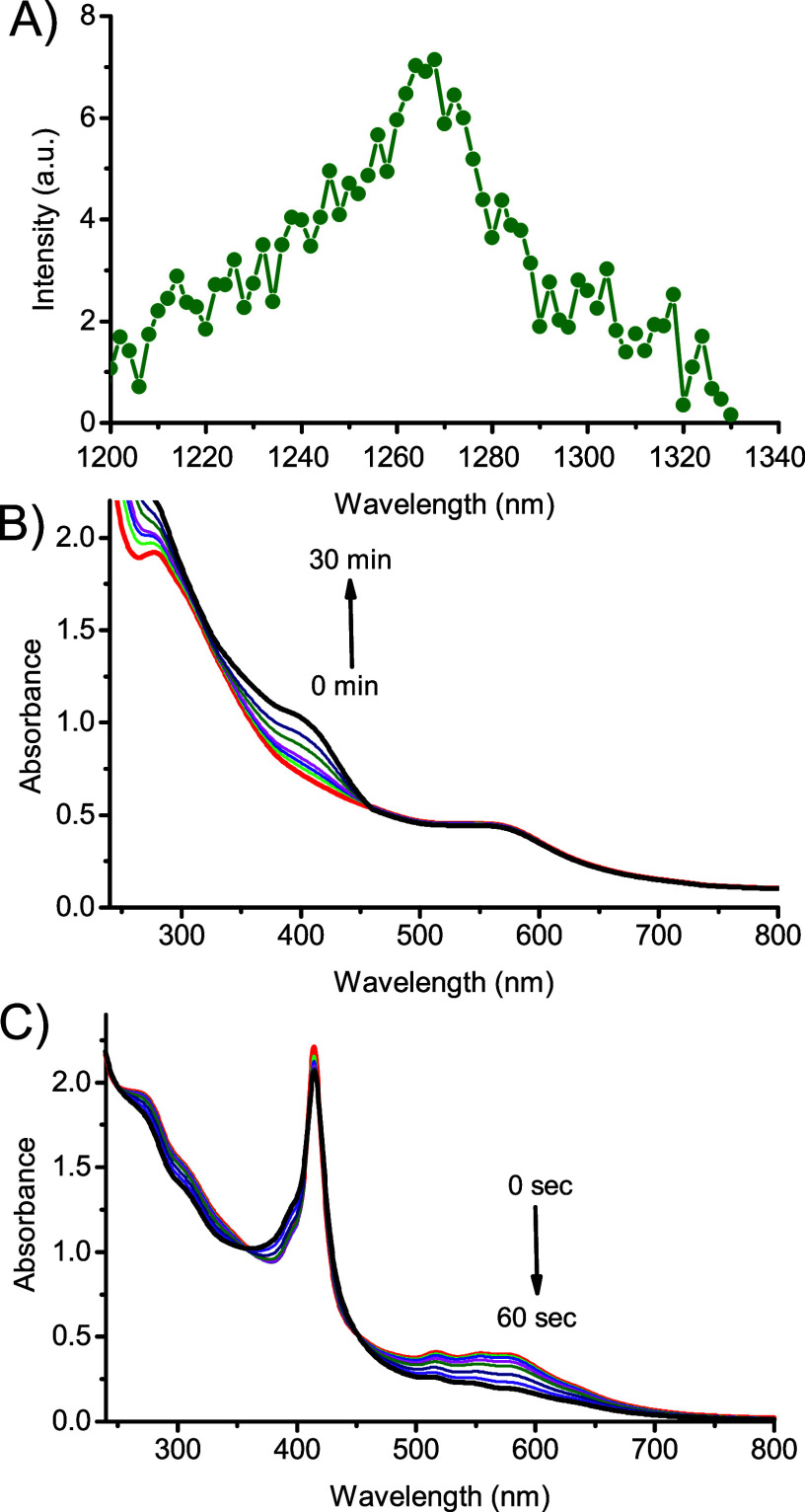
(A) ^1^O_2_ phosphorescence detected under 532 nm light excitation of D_2_O dispersions (1% MeOD) of **NCDs-1** (70 μg mL^–1^). (B) Absorption spectral changes observed under 532 nm light excitation of an air-equilibrated aqueous dispersion (1% MeOH) of **NCDs-1** (70 μg mL^–1^) in the presence of histidine (15 mM) at different irradiation times (from 0 to 30 min). (C) Absorption spectral changes observed under 420 nm light excitation of an air-equilibrated aqueous dispersion (1% MeOH) of **NCDs-1** (70 μg mL^–1^) in the presence of TPPS (5 μM) at different irradiation times (from 0 to 60 s). The arrows in (B,C) indicate the evolution of the spectral profile with the illumination time. *T* = 25 °C.

The fluorescence emission of **NCDs-1** (see Figure 4B) was satisfactory for monitoring its internalization in 9L/LacZ brain cancer cells. Confocal fluorescence microscopy images show that the nanoconjugate mainly localizes outside the nucleus stained with DAPI (blue emission) and mainly in the cytoplasm (Figure 7A). The biological activity of **NCDs-1** was evaluated by preliminary experiments against the same cell lines. The cancer cells were incubated with **NCDs-1** and, for the sake of comparison, with the naked **NCDs**, and then either kept in the dark or illuminated with green light. Figure 7B shows that both **NCDs-1** and **NCDs** are well tolerated in the dark (cell viability *ca.* 80%), accounting for a good biocompatibility of the nanoconstructs (additional images in Figure S6). On the other hand, a considerable reduction in cell viability was observed under illumination. Taking into account that (i) both samples are optically matched at the excitation wavelength (they absorb the same number of photons), (ii) **NCDs** generated ^1^O_2_ with efficiency comparable to **NCDs-1** (Figure S7), and (iii) the photothermal efficiency of the nanoconstructs was below 5%, the higher level of photodynamic inactivation induced by **NCDs-1** cannot be due to a trivial effect. Rather, these findings account for the involvement of a dual-modal mechanism in cell death, more likely due to synergistic/additive photodynamic action in which the NO and ^1^O_2_ simultaneously photogenerated by **NCDs-1** may play a key role.

**Figure 7:**
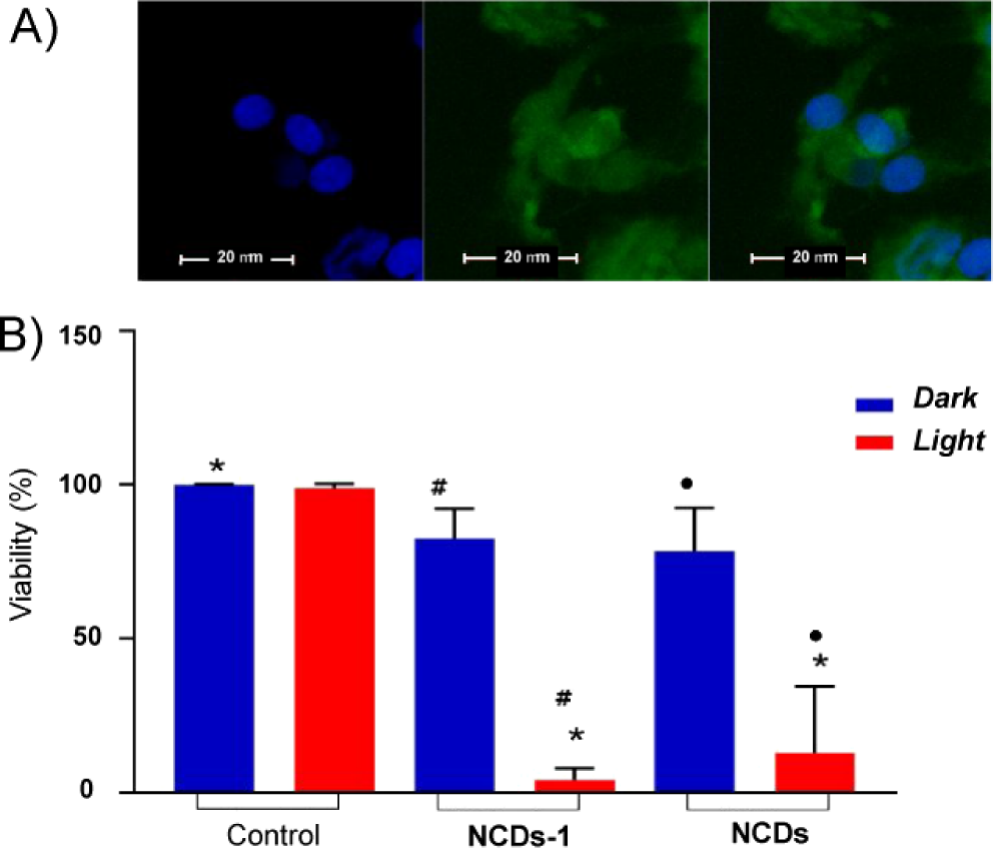
(A) Confocal fluorescence images of 9L/LacZ brain cancer cells incubated 1 h with **NCDs-1** (70 μg mL^–1^) and DAPI observed at λ_exc_ = 488 nm (left panel) and λ_exc_ = 405 nm (center panel) and collecting fluorescence in the range 500–550 nm and 425–475 nm, respectively; the right panel shows the merged images. (B) Cell viability of the same cancer cells incubated 1 h with **NCDs-1** (70 μg mL^–1^) and **NCDs** (17 μg mL^–1^) either kept in the dark or irradiated with green light. *p < 0.001 vs control group (same condition); ●*p* < 0.001 between **NCDs** and **NCDs** under light conditions. #*p* < 0.001 between **NCDs-1** and **NCDs-1** under light conditions.

## 4 Conclusions

We have prepared a nanoconjugate by covalent integration of a blue light-activatable NOPD into the shell of the green light-absorbing **NCDs** scaffold. The **NCDs** core of the resulting nanoconstruct acts as the sole green light-harvesting antenna, permitting the release of NO from the NOPD by an intramolecular photoinduced electron transfer, with a step forward of about 100 nm toward longer and more biocompatible excitation wavelengths. Simultaneously, green light excitation of the nanoconjugate generates ^1^O_2_ by collisional energy transfer with molecular oxygen. To our knowledge, this is the first example of a CDs-based construct generating NO and ^1^O_2_ by single-photon excitation of the CD core with green light. The nanoconjugate (i) internalizes in brain cancer cells, localizing mainly at a cytoplasmic level, (ii) is well tolerated by the cancer cells in the dark, and (iii) induces remarkable cancer cell mortality under green light irradiation by a combined photodynamic action of NO and ^1^O_2_.

## Supplementary Material



## Data Availability

All raw data are available, and the authors will submit them upon request.

## Notes

HRTEM of **NCD-1**; absorption spectra of free **1** and **2**; fluorescence spectra of **NCDs**; photolysis of free **1** with green light and **NCDs-1** with blue light; ^1^O_2_ luminescence of **NCDs-1** and **NCDs**; images of cancer cells; atomic percentages of different species of **NCDs** (PDF)
