# Supplementary material for: N‑Doped Carbon Dot-Based Nanoconjugates with Simultaneous Generation of Nitric Oxide and Singlet Oxygen for Phototherapeutic Applications
Source: ACS Appl Nano Mater. 2025 Jun 16;8(25):13083–91. doi: 10.1021/acsanm.5c02198 (PMC12210281; doi:10.1021/acsanm.5c02198)
Supplement: Supplementary file 1 [file an5c02198_si_001.pdf]

Supporting Information

N-Doped Carbon Dot-Based Nanoconjugates with Simultaneous Generation of Nitric Oxide and Singlet Oxygen for Phototherapeutic Applications

Francesca Laneri,[#†] Cristina Parisi, [#†] Vittoria Andrigo,[§] Juliana Guerra Pinto,[‡] Luciana Cortez Marcolino, [‡] Juliana Ferreira-Strixino,[‡] Marta Maria Natile[§] and Salvatore Sortino[#]*

[#] *PhotoChemLab, Department of Drug and Health Sciences, University of Catania, I-95125, Catania, Italy;*

[‡] *Laboratory of Photobiology Applied to Health, Research and Development Institute, University of Vale do Paraíba, Urbanova I-2911, Brazil*

[§] *ICMATE-CNR Institute of Condensed Matter Chemistry and Technologies for Energy, National Research Council and Department of Chemical Science, University of Padova, 35131 Padova, Italy*

[*] Corresponding author: ssortino@unict.it

[†] Contributed equally

- **Figure S1……………………………………………………………………………S2**
- **Figure S2……………………………………………………………………………S2**
- **Figure S3……………………………………………………………………………S3**
- **Figure S4……………………………………………………………………………S3**
- **Figure S5……………………………………………………………………………S4**
- **Figure S6……………………………………………………………………………S5**
- **Figure S7……………………………………………………………………………S6**
- **Table S1……………………………………………………………………………..S7**

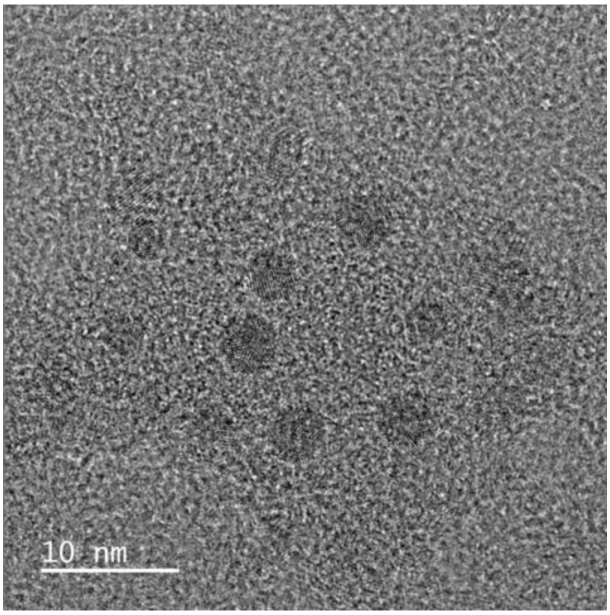

Figure S1. HRTEM of **NCD-1**.

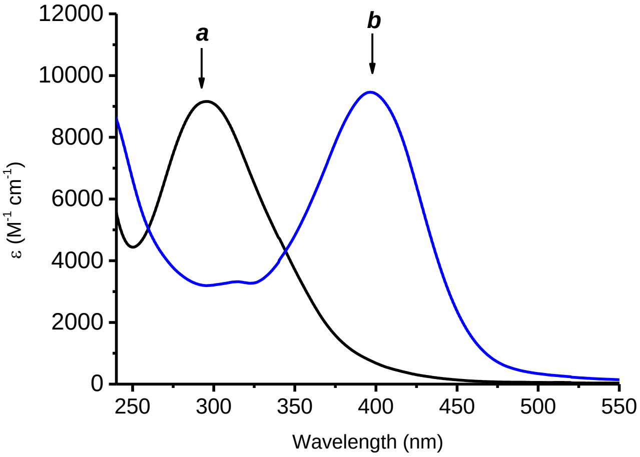

Figure S2. Absorption spectra of **1** (***a***) and of the non nitrosate analogue **2** (***b***).

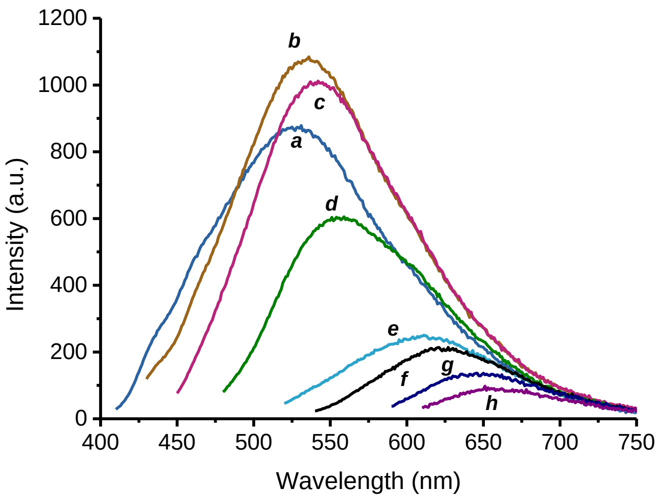

Figure S3. Fluorescence emission spectra of an aqueous dispersion (1% MeOH) of **NCDs** (17 µg mL^{-1}) at different excitation wavelengths: (***a***) = 400 nm; (***b***) = 420 nm; (***c***) = 440 nm; (***d)*** = 470 nm; (***e***) = 510 nm; (***f***) = 530 nm; (***g***) = 580 nm; (***h***) = 600 nm.

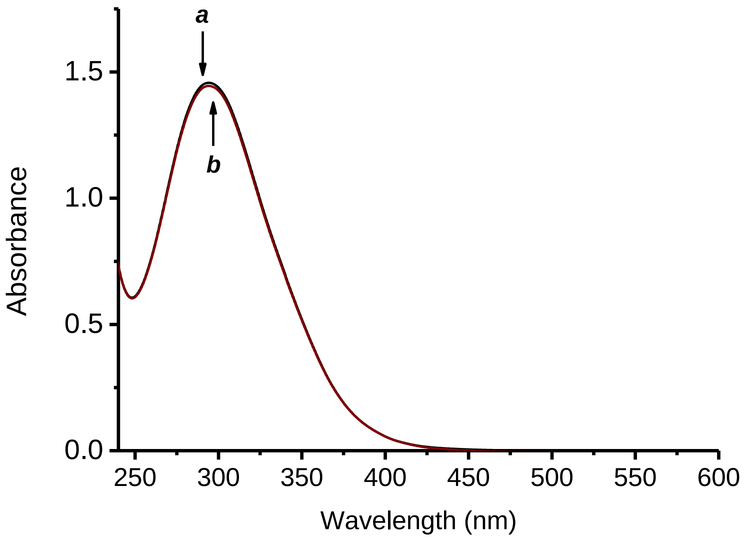

Figure S4. Absorption spectrum of an aqueous solution (1% MeOH) of **1** (53 µg mL^{-1}) before (***a***) and after 2h of irradiation at 532 nm (***b***).

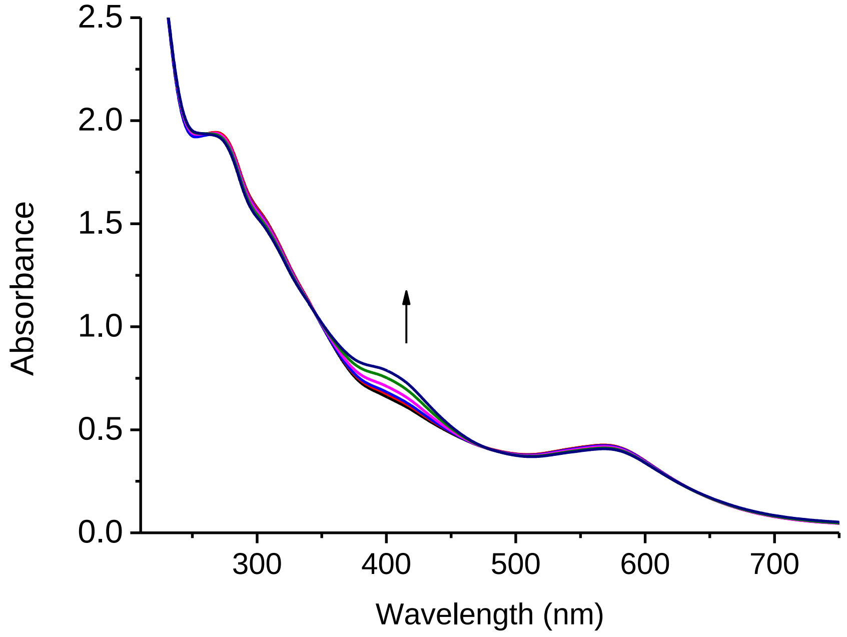

Figure S5. Absorption spectral changes observed upon exposure of an air-equilibrated aqueous dispersion (1% MeOH) of **NCDs-1** (70 µg mL-1) at λ_{exc} = 420 nm at different irradiation times from 0 to 60 sec. The arrow indicates the course of the spectral profile with the illumination time. T = 25 °C.

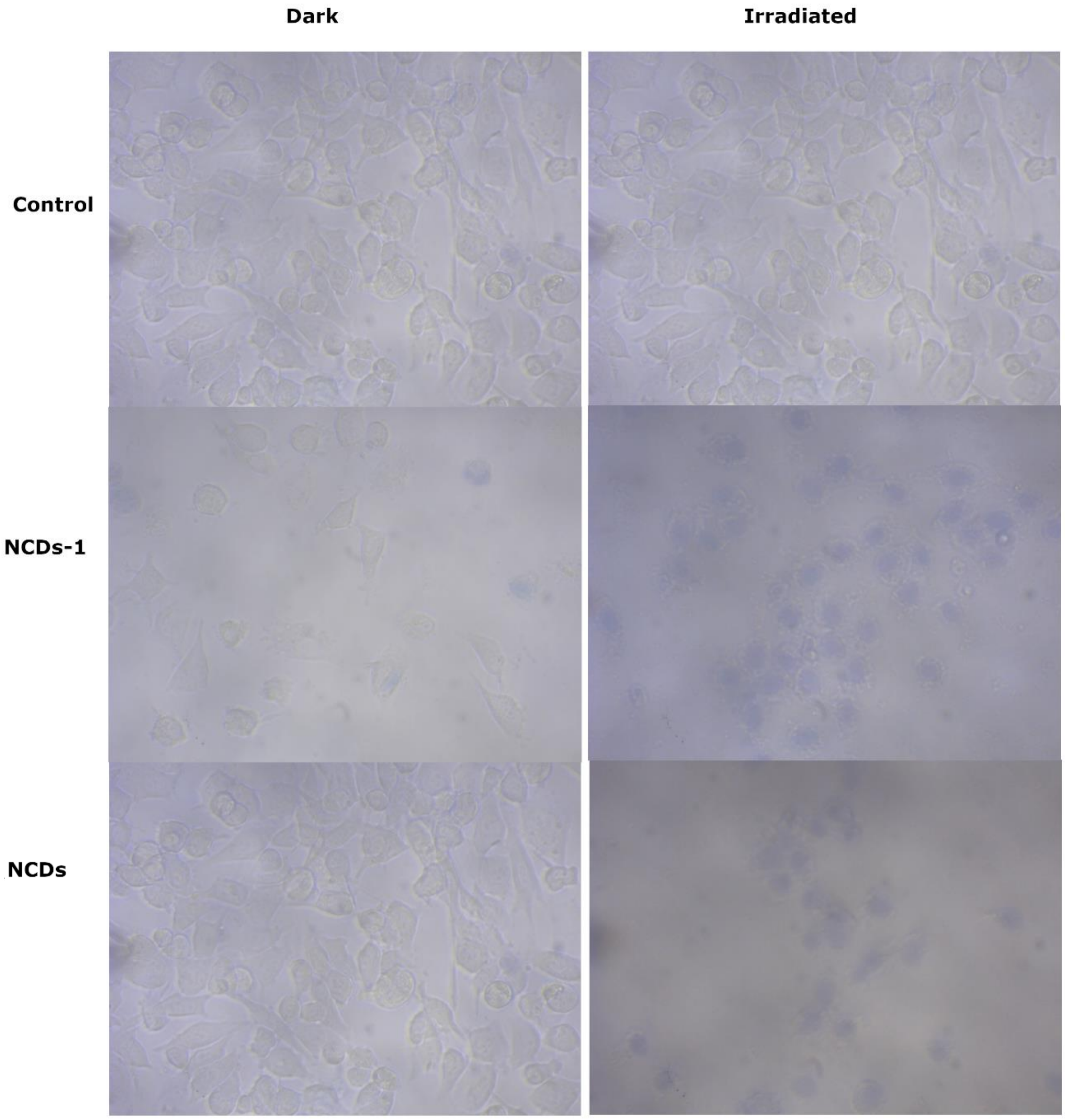

Figure S6. Representative images of 9L/LacZ brain cancer cells incubated 4 h without and with **NCDs-1** (70 µg mL-1) and **NCDs** (17 µg mL-1) either kept in the dark or irradiated with green light.

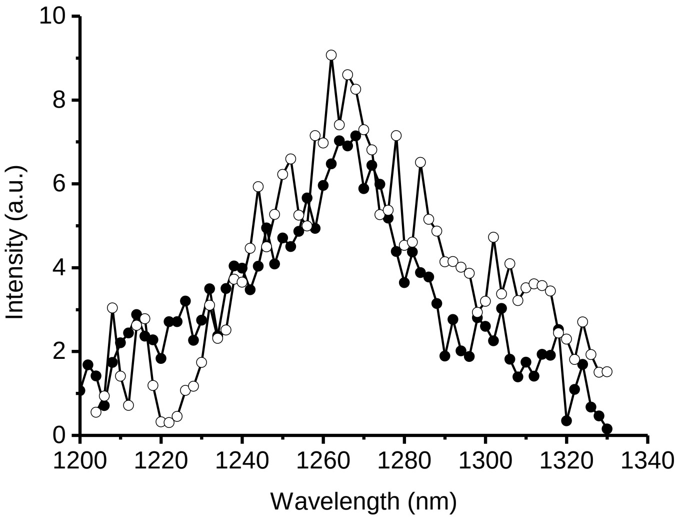

Figure S7. $^1O_2$ luminescence detected upon 532 nm light excitation of optically matched D_2O dispersions (1% MeOD) of **NCDs-1** (●) and **NCDs** (○). Absorbance ~ 0.38 at the excitation wavelength.

Table S1. Summary of different species (eV) and corresponding atomic percentages.

C 1s		N 1s		O 1s	
**BE (eV)**	**Atomic %**	**BE (eV)**	**Atomic %**	**BE (eV)**	**Atomic %**
284.8	38.4	398.6	5.7	530.7	24.3
285.5	22.6	399.6	37.2	531.6	44.9
287.1	25.8	400.1	52.1	533.1	30.8
288.8	13.2	402.1	5.0	--	--
